# Dynamic mechanical interaction between injection liquid and human tissue simulant induced by needle-free injection of a highly focused microjet

**DOI:** 10.1038/s41598-021-94018-6

**Published:** 2021-07-15

**Authors:** Yuta Miyazaki, Masashi Usawa, Shuma Kawai, Jingzu Yee, Masakazu Muto, Yoshiyuki Tagawa

**Affiliations:** Department of Mechanical Systems Engineering, Tokyo University of Agriculture and Technology, Koganei, Japan

**Keywords:** Biomedical engineering, Physics

## Abstract

This study investigated the fluid–tissue interaction of needle-free injection by evaluating the dynamics of the cavity induced in body-tissue simulant and the resulting unsteady mechanical stress field. Temporal evolution of cavity shape, stress intensity field, and stress vector field during the injection of a conventional injection needle, a proposed highly focused microjet (tip diameter much smaller than capillary nozzle), and a typical non-focused microjet in gelatin were measured using a state-of-the-art high-speed polarization camera, at a frame rate up to 25,000 f.p.s. During the needle injection performed by an experienced nurse, high stress intensity lasted for an order of seconds (from beginning of needle penetration until end of withdrawal), which is much longer than the order of milliseconds during needle-free injections, causing more damage to the body tissue. The cavity induced by focused microjet resembled a funnel which had a narrow tip that penetrated deep into tissue simulant, exerting shear stress in low intensity which diffused through shear stress wave. Whereas the cavity induced by non-focused microjet rebounded elastically (quickly expanded into a sphere and shrank into a small cavity which remained), exerting compressive stress on tissue simulant in high stress intensity. By comparing the distribution of stress intensity, tip shape of the focused microjet contributed to a better performance than non-focused microjet with its ability to penetrate deep while only inducing stress at lower intensity. Dynamic mechanical interaction revealed in this research uncovered the importance of the jet shape for the development of minimally invasive medical devices.

## Introduction

Mechanical interaction between fluid and body tissues, such as blood and tunica intima in blood vessels, cerebrospinal fluid and meninges in the brain, etc.^1–10^, is essential to biomedical engineering and related fields. Especially for needle-free injectors which deliver liquid drugs through the skin using high-speed liquid jets, the possible damage and delivery efficiency are greatly influenced by the interaction between the liquid jet and the body tissue. The needle-free injectors have undergone rapid development due to the demand for minimally invasive systems which can reduce the risk of accidents and trypanophobia (needle phobia) caused by injection via standard hypodermic needles (penetration depth: 5–8 mm)^11–20^. Although some needle-free injectors have been released to the market, a previous study pointed out that the pain induced by these injectors is significantly greater than that by the conventional needle injection, while the efficacy is less^21^. Most of the existing needle-free injectors generate non-focused liquid jets, the tip of which has a diameter larger than the nozzle and spreads out as the jet travels^11,12^. This causes a wide area of the skin to be exposed to the liquid jet during the injection, causing severe damage to the skin, and hence pain to the patients^14^. Besides, most of the studies which evaluated pain intensity induced by the needle-free injectors are in-vivo clinical trials on human bodies which were carried out through survey^22–26^ and the survey results often depend on subjective assessment.

This article investigated the fluid–tissue interaction as a quantitative and objective evaluation on the stress intensity and the damage caused by the needle-free injection of a highly “focused” microjet, the tip of which has a diameter much smaller than the nozzle and does not disperse as the jet travels^27,28^. Several studies have reported the potential of the focused microjet for the development of a needle-free injector with reduced pain and improved delivery efficiency^29–34^. An ex-vivo study of the focused microjet has been carried out on hairless rat skin by our research group^35^. The study visualized the penetration dynamics of the focused microjet and investigated the relationship between the penetration depth and the width of the penetration site. As the next step to elucidate the fluid–tissue interaction during a needle-free injection, this study evaluated the unsteady mechanical stress field induced by the focused microjet in body tissue simulant using a high-speed photoelastic measurement system^36,37^. In addition, the stress field induced by the focused microjet was analyzed and compared with that by a non-focused microjet and a standard hypodermic needle (penetration depth: 5–8 mm)^38–41^. While in previous studies^42,43^, the focus was on the injection pattern shape obtained by absorption contrast of the injected dye, our polarization-based method provides essential insights on the inner stress in materials from light polarization contrast.

## Experimental methodology

### Photoelasticity and stress field measurement

**Figure 1:**
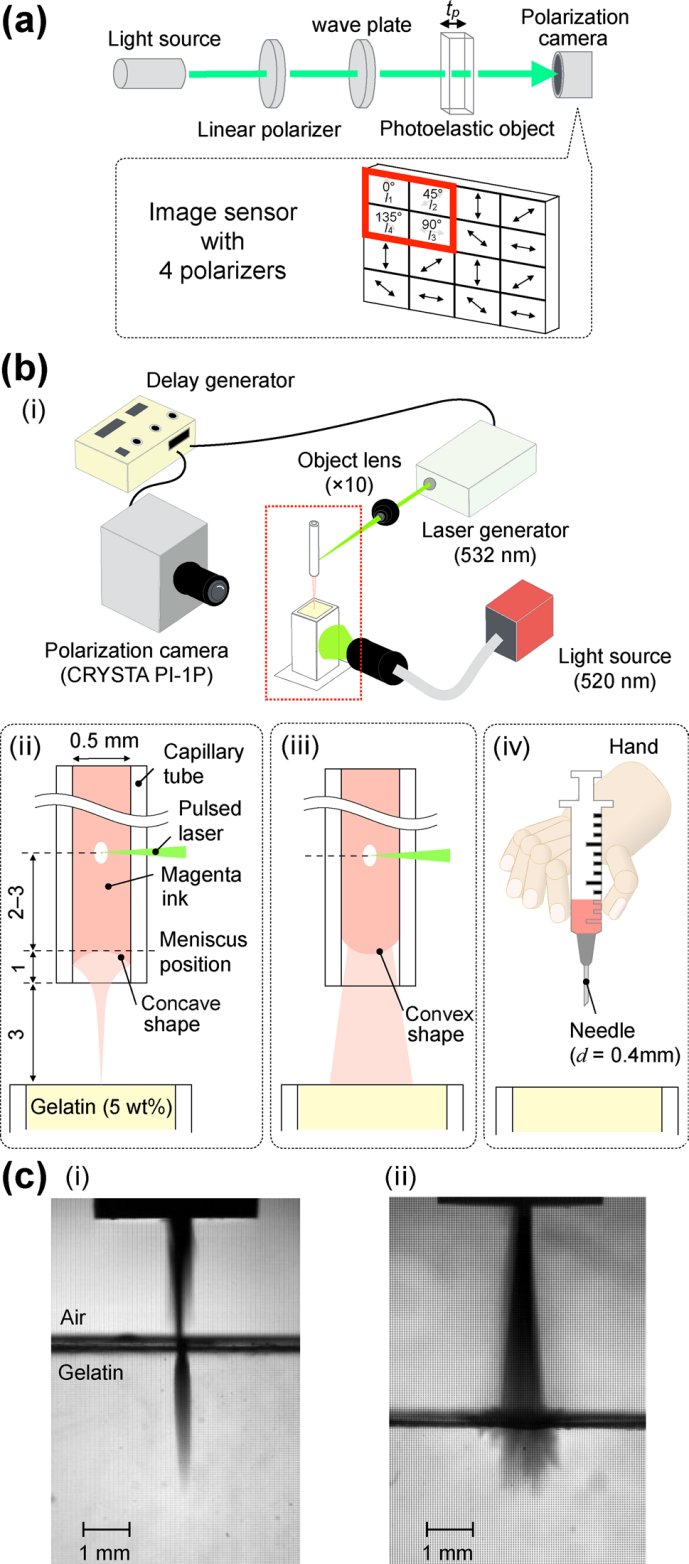
(**a**) Schematic diagram of polarization measurement with high-speed polarization camera. (**b**) (i) Schematic diagram of experimental setup for the photoelastic measurement of stress field induced by (ii) focused microjet, (iii) non-focused microjet, and (iv) needle penetrations. (**c**) Ejection shapes of (i) focused microjet and (ii) non-focused microjet.

The principle of the photoelastic measurement of our system is described in this subsection. To visualize the dynamic stress field in the body tissue simulant as a result of the interaction with the microjets, photoelastic measurement using a high-speed polarization camera (CRYSTA PI-1P, Photron Ltd., maximum frame rate: 1,550,000 f.p.s.) was employed. The optical setup for the photoelastic measurement is shown in Fig. 1a. In this figure, a photoelastic object is placed on the optical axis between the camera and a circular polarized backlight (520 nm, SLG-55-G, REVOX Inc.). Under stress, the photoelastic object splits the transmitted circular light into an ordinary ray and an extraordinary ray. The effective refractive indices of these two rays are different from one another, resulting in an optical phase difference^44^. The optical phase difference $\varDelta$ is linearly proportional to the difference in the internal principal stresses $\sigma _d$^45^ as shown in the following equation:

1 $$\begin{aligned} \varDelta = C t_{p} { \sigma }_{ d }, \end{aligned}$$

where *C* is the stress-optical coefficient and $t_{p}$ is the thickness of the photoelastic object along the optical axis.

The special feature of the high-speed polarization camera used is the pixelated polarizer array that enables it to capture the unsteady photoelasticity^36^. As shown in Fig. 1a, the array consists of four adjacent polarizers (2$\times$2), arranged in four different orientations at 0$^\circ$, 45$^\circ$, 90$^\circ$, and 135$^\circ$. With this feature, the camera is able to simultaneously capture the intensity of the incident light at four different orientations ($I_1$ at 0$^\circ$, $I_2$ at 45$^\circ$, $I_3$ at 90$^\circ$, and $I_4$ at 135$^\circ$). Thus, the instantaneous phase difference $\varDelta$ and the principal azimuthal angle $\varphi$ can be calculated from these four different intensities of the incident wavelength of light $\uplambda$^36^. Note that the phase difference $\varDelta$ is obtained by the integration along the optical axis of the camera within the photoelastic object.

2 $$\begin{aligned} {\varDelta }= & {} \left( \frac{\uplambda }{2 \pi } \right) \sin ^{ -1 }{ \frac{ \sqrt{ { \left( { I }_{ 3 }-{ I }_{ 1 } \right) }^{ 2 }+{ \left( { I }_{ 2 }{ -I }_{ 4 } \right) }^{ 2 } } }{ { I }_{ 1 }+{ I }_{ 2 }+{ I }_{ 3 }+{ I }_{ 4 } } } \end{aligned}$$

3 $$\begin{aligned} \varphi= & {} \frac{1}{2}\tan ^{ -1 }{ \frac{ { I }_{ 3 }-{ I }_{ 1 } }{{ I }_{ 2 }-{ I }_{ 4 }}} \end{aligned}$$

### Experimental setup

In our photoelastic measurement system, gelatin gel was used as the body tissue simulant. The concentration of the gelatin gel (Gelatin from porcine skin, Sigma-Aldrich Co. LLC.) was fixed at 5 wt$\%$ to simulate the viscoelasticity of the human tissue^16,30^. Note that multiple materials with different elastic properties are necessary to simulate the complex properties of human skin^46^. Since the ability of the focused microjet to penetrate human and animal skin has been proven^30,35^, this study focused on simulating the induced stress in the human tissue.

Figure 1b (i) shows the schematic of the experimental setup. The gelatin gel block was placed inside a plastic container (8 mm $\times$ 8 mm $\times$ 45 mm) with the top and bottom interfaces opened to the atmosphere. Microjet was ejected vertically downwards towards the gelatin surface, which was 3 mm away from the edge of the capillary tube (see Fig. 1b (ii)). To induce the microjet, a pulsed laser beam with a wavelength of 532 nm (Nd:YAG Laser NANO, Litron Laser Ltd.) was focused onto a point inside the liquid in the capillary tube (inner diameter: 0.5 mm, outer diameter: 3 mm) through an objective lens (magnification: 10$\times$, NA: 0.25, SLMPLN 10$\times$, Olympus Co.). The laser energy was estimated to be 1.9 mJ based on the results of the experiment data from a previous work^27^. The position where the laser focused on was 2–3 mm away from the meniscus position (1 mm above the edge of the capillary tube). When the pulsed laser struck the liquid inside the capillary tube, the laser-induced bubble expanded quickly, inducing a shockwave^47^. As a result, a microjet is ejected^48^. As for the jet volume, the maximum liquid volume that can be ejected from the capillary tube is estimated to be about 0.5 μL.

The shape of the ejected microjet (focused or non-focused) depends on the shape of the initial meniscus of the liquid. As shown in Fig. 1b (ii)(iii), when the initial meniscus has a concave shape (contact angle < 90$^\circ$), the flow of the liquid around the meniscus is converged, which induces the ejection of a focused microjet^27^. On the other hand, when the initial meniscus has a convex shape (contact angle > 90$^\circ$), a non-focused microjet is ejected^27^. The liquid used contained magenta ink which enhanced the energy absorption from the green pulsed laser. The penetration into the gel by both focused and non-focused microjets was recorded by the polarization camera at 25,000 f.p.s., while the needle penetration (27G$\times$1" (": inch), outer diameter: 0.4 mm, TERUMO Co.) was recorded at 500 f.p.s. The needle injection was performed by an experienced nurse (see Fig. 1b (iv)). For all three penetrations reported in this article, the spatial resolution is 13–15 μm/pixel.

Snapshots when a focused and a non-focused microjets touches the gelatin are shown in Fig. 1c (i), (ii), respectively. The tip diameter of the focused microjet is in the order of tens of micrometers, which is much smaller than the nozzle diameter, 500 μm. In contrast, the tip diameter of the non-focused microjet is larger than the nozzle diameter. Note that the difference in the jet shape is due to the shape of the initial meniscus inside the capillary tube^27^.

## Result and discussion

### Stress intensity field

**Figure 2:**
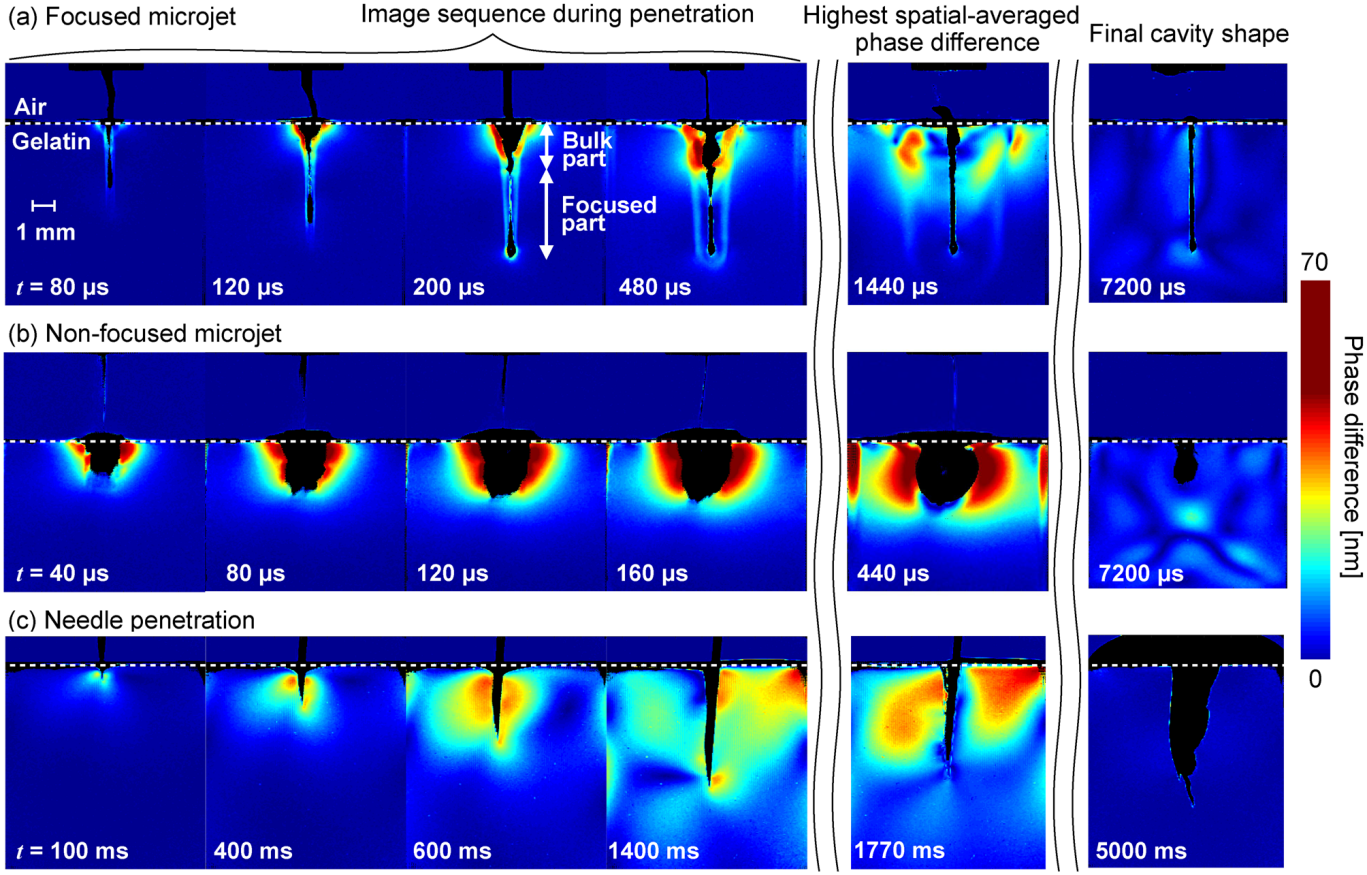
Image sequence of stress fields induced by the penetration of (**a**) the focused microjet, (**b**) the non-focused microjet, and (**c**) the needle. Note that the relative intensity distribution of a phase difference field is proportional to the intensity distribution of a stress field.

The temporal evolution of the stress intensity field induced by the focused microjet, the non-focused microjet, and the needle penetration was visualized and discussed in this section. The image sequence of the visualized result is shown in Fig. 2. In this figure, the black-masked area represents the cavity generated by the microjets and the needle. Moreover, the red area represents phase difference of high intensity, while the blue area represents phase difference of low intensity. As shown in Eq. (1) in "Photoelasticity and stress field measurement" section, the relative intensity distribution of the phase difference field is proportional to that of the stress field. Therefore, phase difference of high intensity in a certain area indicates stress of high intensity in the same area. For the quantitative comparison of the stress intensity over the stress field induced by the microjets and the needle, spatial averaged values of phase difference were calculated by considering the area which includes every pixel below the gelatin surface, but excludes the area of the cavity generated by the microjets and the needle.

The development of the stress field surrounding the cavity generated by the focused microjet at a velocity of 198 m/s was visualized through the image sequence shown in Fig. 2a. In the case of the microjets, the initial time of the image sequence (0 μs) is defined as the time when the liquid of the microjets just touches the gelatin surface. Up until ${ t}$ = 200 μs, the shape deformation of the cavity induced by the injection of the focused microjet was significantly different between the area near the tip deep inside the gelatin and the area near the gelatin’s surface. The cavity was narrow in the area where the tip was deep inside the gelatin while it rapidly expanded over time in the area near gelatin surface. The narrow cavity near the tip is designated as the focused part, while the rapidly expanding cavity near the gelatin surface is designated as the bulk part (see snapshot at 200 μs). It was observed that the bulk part shrank significantly as compared to the focused part after the maximum expansion of the cavity. In addition, the stress intensity in the area surrounding the focused part was lower than that of the bulk part. Even after the injection stopped at 200 μs, the stress wave continued to propagate in the gel (see snapshots at 200–1440 μs). Here, the measured propagation speed of the stress wave was about 1.5 m/s, which is same as the reported velocity of the shear wave in 5 wt$\%$ gelatin gel (1.52 ± 0.10 m/s)^49^. After the phase difference reached the highest spatial averaged value at 1440 μs, the shape of the cavity remained almost unchanged, while the stress wave dissipated over time (see 7200 μs).

For comparison with the focused microjet, the development of the stress field surrounding the cavity generated by the injection of a non-focused microjet of the same liquid ejection volume and the same input laser energy was visualized and is shown in Fig. 2b. Note that the non-focused microjet has a velocity of 150 m/s, which is slower than that of the focused microjet in Fig. 2a. Due to the diffusive shape of the microjet, the cavity induced by the non-focused microjet did not have a focused part, which is different from that by the focused microjet. Instead, a rapid expansion of the cavity, which is similar to the bulk part induced by the focused microjet, was observed (see snapshots at 40–160 μs). In addition, it was significantly larger than that of the focused microjet. Since the ejection volume was the same, this indicates that the rapidly expanding cavity of the non-focused microjet (the black-masked area) contained a larger amount of air as compared to that of the focused microjet. When the phase difference reached the highest spatial averaged value at 440 μs, the cavity shrank significantly as the air escaped from the cavity. Finally, after all the air escaped from the cavity, there was only a small cavity remained (see snapshot at 7200 μs). This significant shrink of the cavity, which also happened in the bulk part for the case of the focused microjet, was caused by the elastic rebound of the gel. The penetration depth of the non-focused microjet was not as deep as that of the focused microjet, which is evident from the final snapshot at 7200 μs. Without inducing a focused part, the rapid expansion at the bulk part is almost only an elastic deformation, and hardly contributes to the injection of the liquid. Therefore, the focused part induced by the focused microjet is essential for the effective injection of the liquid.

For the comparison with the conventional method of liquid injection, the stress field induced by needle injection performed by an experienced nurse was visualized and is shown in Fig. 2c. Note that the stress field was induced by the interaction between the solid needle and tissue simulant instead of fluid–tissue interaction for the cases of the microjets. The process can be divided into three stages: penetration, liquid injection, and withdrawal. In the penetration stage, the needle touched the gelatin’s surface at 0 ms and penetrated until 1400 ms (see snapshots at 100–1400 ms). The snapshots at 1770 ms and 5000 ms show the liquid injection stage and withdrawal stage, respectively. The stress intensity was higher around the needle, and the distribution was wide and asymmetric (see snapshots at 600–1770 ms). This is attributed to the movement of the needle induced by the nurse, instead of the propagation in the form of waves in the case of the focused microjet. Note that overflow of the injected liquid was observed during needle injection. In real-life applications, such overflow does not occur due to the presence of human skin and the poroelasticity of human tissue^50^ which helps to absorb the liquid drugs.

### Stress vector field

**Figure 3:**
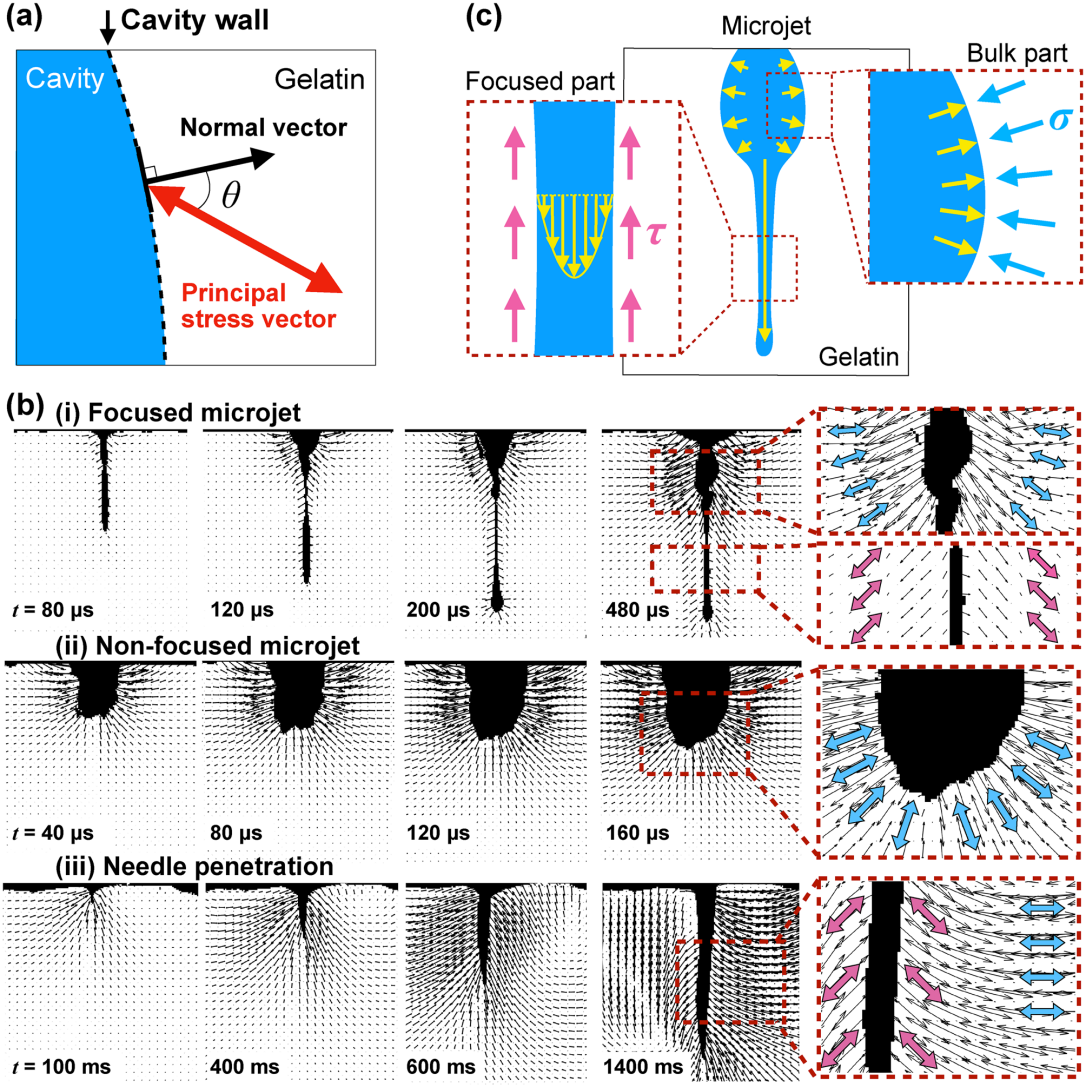
(**a**) Illustration of principal stress vector and normal vector (moving direction of cavity wall). (**b**) Stress vector fields induced by the penetration of (i) the focused microjet, (ii) the non-focused microjet and (iii) the needle. The black arrows indicate the direction between the principal stress vector and the normal vector of the cavity wall. The blue or pink arrows show the principal stresses which are dominated by either compressive stress or shear stress, respectively. (**c**) Conjecture of flow direction and stress vector induced by the focused microjet. The yellow vector indicates the flow direction of the liquid. The dominance of different types of stress vectors is related to the flow direction of the liquid.

To understand the fluid–tissue interaction during the needle-free injection, i.e. the influence of the injected liquid on the induced cavity, it is necessary to determine whether compressive stress (normal stress) or shear stress is in dominance acting on the tissue, through visualizing the stress vector field. In this section, such dominating stress in the stress field of the gelatin induced by the interaction during the penetration of the microjets and needle is discussed. With our system, the principal stress vector field can be visualized by using Eqs. (2) and (3). To determine whether compressive stress or shear stress is dominant against the cavity wall, the angle between the principal stress vector and the normal vector of the cavity wall is defined as the angle $\theta$, as shown in Fig. 3a. The dominance of compressive stress $\sigma$ or shear stress $\tau$ can be estimated from the angle $\theta$ using the following equation^51^.

4 $$\begin{aligned} \theta = \frac{1}{2}\sin ^{-1}{\frac{\tau }{\sqrt{\tau ^2+\sigma ^2}}} \end{aligned}$$

According to Eq. (4), $\theta$ is close to $\pi /4$ (45$^\circ$) when the shear stress is dominant, while $\theta$ is close to 0 (0$^\circ$) when the compressive stress is dominant.

The stress vector fields of the principal stress resulted by the interaction with focused microjet, non-focused microjet, and needle penetration were visualized and are shown in Fig. 3b, in which the vector length is proportional to the magnitude of the phase difference. The vector field induced by the focused microjet shows that the stress vector was continuously induced around the tip of the cavity and moved in the spanwise direction (see snapshots at 80–480 μs of Fig. 3b (i)), confirming the observations on Fig. 2a. Remarkably, it also revealed the dominance of different types of stress in different parts of the induced cavity (see snapshot at 480 μs). Around the focused part, the principal stress vectors shown as pink arrows have $\theta$ of approximately 45$^\circ$, indicating that shear stress was dominant. In contrast, around the bulk part, the vectors shown by the blue arrows are normal to the cavity wall ($\theta$ = 0$^\circ$), indicating that compressive stress was dominant. The length shows that the stress intensity around the bulk part was higher than that of the focused part. Moreover, the compressive stress was dominant in areas with high stress intensity, while shear stress was dominant in areas with low stress intensity. Apart from differences in stress intensity, dominance of different types of stress is also characterized by different flow directions. For clearer explanation, a conjecture of the physical representation is shown Fig. 3c. At the focused part, the liquid flows in the direction parallel to the cavity wall, inducing shear stress. Whereas, at the bulk part, the liquid flows in the direction normal to the cavity wall, inducing compressive stress. Thus, the focused part of the cavity dominated by shear stress, helps the injected liquid to flow deep into the gel at low intensity.

In the case of the non-focused microjet (see Fig. 3b (ii)), compressive stress with high intensity was dominant around the whole induced cavity, indicating liquid flow in the direction normal to the cavity wall. This phenomenon is similar to the bulk part of the cavity induced by the focused microjet. However, shear stress was not induced without inducing a focused part, preventing the cavity from penetrating deep into the gel. Instead, dominant compressive stress with high intensity was induced around the cavity and widely distributed in the gel.

Previous studies^30,38^ have assumed that viscous shear stress acts on the jet-gelatin interface due to shear flow, and that a vertical reaction force acts on the projected area of the jet shown by the dashed line in the lower left figure. These considerations are consistent with the results of the present study, in which the stress was evaluated in a vector field. Therefore, the visualization of the stress field in this study provides experimental evidence for the models of the previous studies.

As for the conventional method of drug delivery, where the vector field is induced by needle penetration was visualized and shown in Fig. 3b (i). The distribution was asymmetric and consisted of both shear stress and compressive stress due to manual movement of the needle by the nurse. Note that the shear stress here was induced by the friction between the needle and the cavity wall, instead of the injected liquid in the cases of microjets.

In summary, the focused part of the cavity induced by the focused microjet enabled the injected liquid to flow deep into the tissue with shear stress at low intensity. This is the remarkable feature of the interaction between the focused microjet and the tissue simulant, which is attributed to the focused shape of the microjet tip. Such feature is essential for the development of a minimally invasive drug delivery mechanism.

### Detailed analysis on stress intensity field

**Figure 4:**
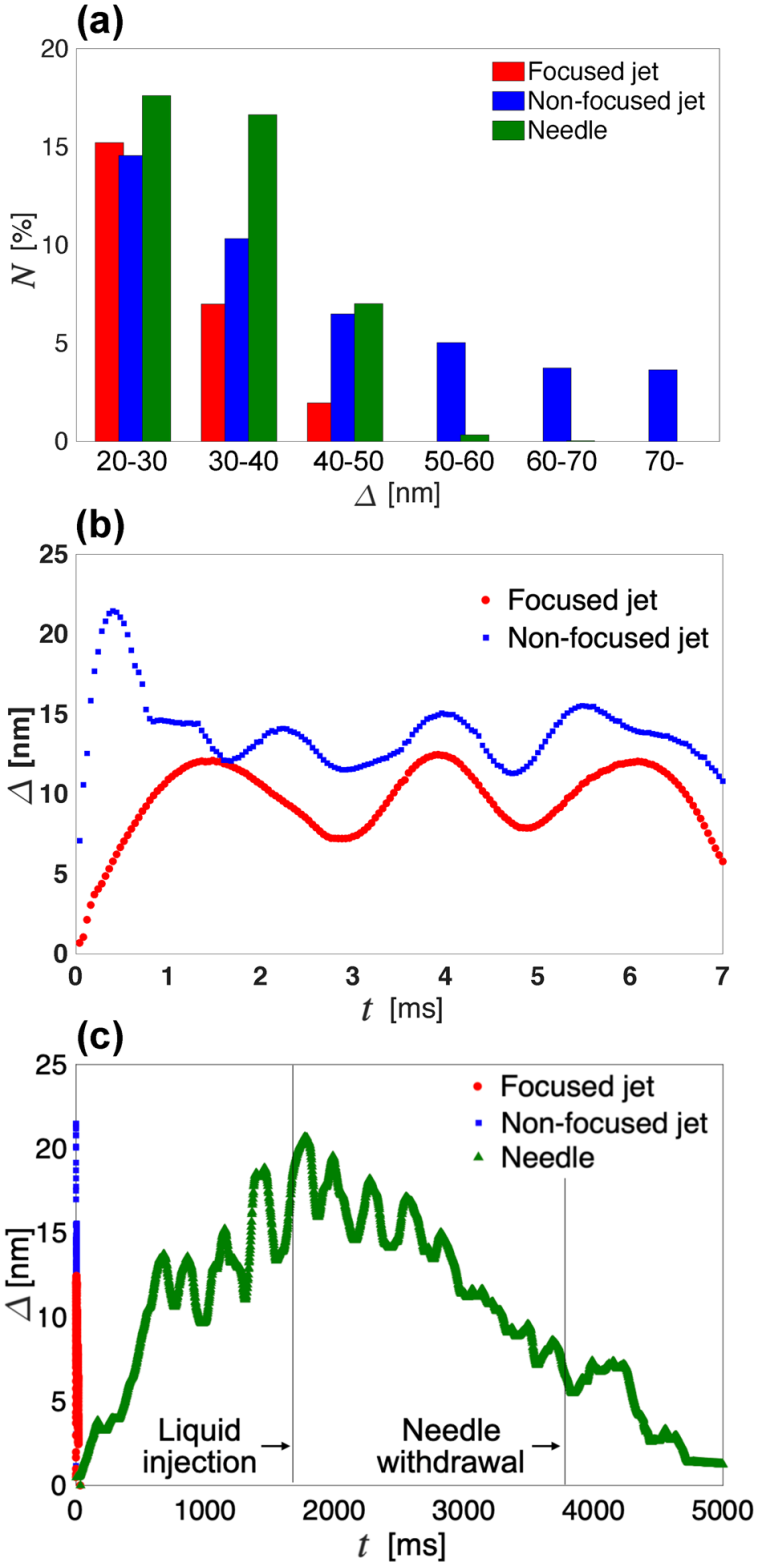
(**a**) Histogram of area percentage *N* of different ranges of phase difference ${\varDelta }$ in the whole image at the time of highest spatial averaged phase difference. The red, blue, and green bars show the result of the focused jet, non-focused jet, and needle respectively. (**b**–**c**) Spatial averaged stress versus time *t* for comparison between (b) the focused and non-focused microjets, (**c**) the microjets and the needle penetration. The red, blue, and green plots show the spatial averaged stress of the focused microjet, the non-focused microjet, and needle injection, respectively.

As a further analysis on the stress intensity field shown in Fig. 2, the distribution of stress intensity over the total area of the gelatin (area under the white dotted line) was quantified. The area percentage *N* with intensity within the respective ranges of measured $\varDelta$ is presented in Fig. 4a. From Fig. 2a, b, at the time of highest spatial averaged phase difference $\varDelta$, stress induced by the non-focused microjet (*t* = 440 μs) had a higher intensity and a wider distribution than that of the focused microjet (*t* = 1440 μs).

The percentage of the area *N* where the phase difference exceeded 40 nm was only 2$\%$ for the case of the focused microjet, while in contrast, it was more than 18$\%$ for the case of the non-focused microjet.

This shows that the stress induced by the focused microjet had lower intensity and the distribution of high intensity was significantly smaller than that of the non-focused microjet, even though the ejection volume and the input energy were the same. This is because the focused part of the cavity resulting from the interaction with the focused microjet played an important role in preventing the generation of stress of high intensity. Therefore, as a minimally invasive method for drug delivery into human tissue, the focused microjet is more effective than the non-focused microjet because of its deeper penetration, lower stress intensity, and a smaller area of stress distribution.

The comparison between the snapshots of the highest spatial averaged phase difference of the focused microjet at 1440 μs and the needle penetration at 1770 ms was also quantified and is shown in Fig. 4a. As mentioned previously, the phase difference exceeding 40 nm was only 2$\%$ for the case of the focused microjet, whereas it was more than 7$\%$ for the case of the needle penetration. Thus, this wide area of high stress intensity can be avoided with the use of the focused microjet.

A comparison on the temporal evolution of the spatial averaged phase difference was carried out and shown in Fig. 4b–c. In Fig. 4b, soon after the injection of the non-focused microjet, the stress intensity showed a sudden increase (*t* = 440 μs) which was about double of that of the focused microjet. These observations can be explained with respect to the time of the formation of the bulk part. As shown in Fig. 2, bulk part formed almost immediately after the injection of the non-focused microjet (see *t* = 40 μs), causing the sudden increase of stress intensity. As for the focused microjet, the bulk part formed after the focused part (see *t* = 120 μs), preventing the sudden increase of the stress intensity. Moreover, the formation of the focused part also suppressed the peak of the stress intensity. Note that for both microjets, the stress intensity oscillated at the order of milliseconds, which was due to the propagation and reflection of the shear wave at the wall of the gel container. As for the case of needle penetration (see Fig. 4c), the duration of the induced stress intensity was in the order of seconds, which was much longer than that of the microjets. The stress intensity, in the case of needle penetration, increased before liquid injection stage for the duration of approximately 2000 ms due to penetration. The stress intensity decreased from the beginning of the liquid injection stage, until approximately 1000 ms after withdrawal of the needle. Note that the oscillation of stress intensity was induced by the hand movement of the nurse. Thus, the duration of induced stress during a needle-free injection using a microjet is significantly shorter than that of conventional needle injection.

## Conclusion

An objective and quantitative assessment of the performance of the focused microjet as a needle-free injector was carried out by evaluating the mechanical stress induced by the injection liquid in a body tissue simulant. For this purpose, the intensity and the vector field of phase difference, which is proportional to stress, induced by the focused microjet, non-focused microjet, and needle penetration were visualized and discussed.

The damage caused by all three types of injectors was evaluated based on the intensity, distribution, and temporal evolution of the induced stress. The stress field induced by the focused microjet showed a significantly lower intensity and a smaller area of distribution than that by the non-focused microjet, despite the similar amount of ejection volume and input energy. The relevant results imply that the focused part of the cavity induced by the focused microjet contributed to the reduction of the stress intensity. In case of conventional needle injection, although the induced intensity was not as high as that of the non-focused microjet, the duration when the stress intensity was induced was much longer than that of both microjets due to the penetration and withdrawal of the needle.

The type of dominating stress acting on the tissue resulted by the fluid–tissue interaction with the injectors was determined and evaluated based on the stress vector field. The visualization of the stress vector field induced by the focused microjet shows that shear stress was dominant around the focused part which penetrated deep into the gelatin while compressive stress was dominant around the bulk part which was near the gelatin surface. The cavity induced by the non-focused microjet exhibited similarity with the bulk part of the cavity induced by the focused microjet, which was dominated by compressive stress and did not penetrate deep into gelatin. Thus, the induced focused part was the key factor which contributed to the efficacy of the focused microjet in penetrating and delivering liquid deep into the gelatin. In the case of the conventional needle penetration, owing to the hand movement of the experienced nurse, both compressive and shear stresses were observed over a wide area, and their distribution was asymmetric.

The fluid–tissue interaction during the injection of a focused microjet shows its great potential to be developed into a safe and efficacious needle-free injector, which can be used to penetrate and deliver drugs deep into the human tissue with minimal mechanical stress. The method implemented in this study which successfully visualized the unsteady mechanical stress field induced by the fluid–tissue interaction during needle-free injection is also applicable for various stress-driven phenomena found in the field of soft matter, physics, biology, medical science, mechanical engineering, among others.
